# Zinc Protoporphyrin Is a Reliable Marker of Functional Iron Deficiency in Patients with Inflammatory Bowel Disease

**DOI:** 10.3390/diagnostics11020366

**Published:** 2021-02-21

**Authors:** Eleni Leventi, Aysegül Aksan, Carl Thomas Nebe, Jürgen Stein, Karima Farrag

**Affiliations:** 1 Klinik für Gastroenterologie, Diabetologie und Infektiologie, Klinikum Hanau, 63450 Hanau, Germany; lena.leventi@gmail.com; 2 Interdisziplinäres Crohn Colitis Centrum Rhein-Main, 60594 Frankfurt am Main, Germany; ayseguel.aksan@ernaehrung.uni-giessen.de (A.A.); kfarrag@khs-ffm.de (K.F.); 3 Institute of Nutritional Science, Justus-Liebig University, 35392 Giessen, Germany; 4 Facharztpraxis für Laboratoriumsmedizin, 68161 Mannheim, Germany; info@haema-labor.com; 5 DGD Kliniken Sachsenhausen, Teaching Hospital of the Goethe-Universität, 60594 Frankfurt am Main, Germany

**Keywords:** anemia, inflammatory bowel disease, iron deficiency, zinc protoporphyrin

## Abstract

Iron deficiency (ID) is a common manifestation of inflammatory bowel disease (IBD), arising primarily due to chronic inflammation and/or blood loss. There is no gold standard for ID diagnosis, which is often complicated by concomitant inflammation. Zinc protoporphyrin (ZnPP) correlates with parameters of iron homeostasis and has been identified as a promising marker for ID, irrespective of inflammation. We investigated the diagnostic performance of ZnPP in ID, iron deficiency anemia, anemia of chronic disease and mixed anemia in a cross-sectional study in 130 patients with IBD. Different parameters were compared by receiver operator characteristic (ROC) analysis as detectors of iron-restricted erythropoiesis (IRE). IRE was detected in 91 patients (70.0%); fifty-nine (64.8%) had absolute ID and 23 (25.4%) functional ID. When inflammation was present, ZnPP was a more reliable sole biomarker of IRE than MCV, transferrin saturation (TSAT) or ferritin (AUC; 0.855 vs. 0.763, 0.834% and 0.772, respectively). The specificity of TSAT was significantly lower than ZnPP when inflammation was present (38% vs. 71%, respectively). We conclude that ZnPP is a reliable biomarker of functional ID in patients with IBD and more dependable than ferritin or TSAT, which are influenced by chronic inflammation. We propose that ZnPP may also have utility in patients with other chronic diseases.

## 1. Introduction

Iron deficiency anemia (IDA) is recognized as one of the most common complications and extraintestinal manifestations of inflammatory bowel disease (IBD). Depending on the population studied (e.g., in- or outpatients, active or quiescent disease, severe or milder disease), anemia is estimated to affect between 13 and 90% of patients with IBD [1,2,3,4].

ID in IBD presents either as absolute ID (AID) or as functional ID (FID). AID arises primarily due to disease-induced malnutrition and (usually chronic) blood loss, whereas FID is caused by impaired gastrointestinal iron absorption and/or impaired release of storage iron due to chronic inflammation or bowel resection (especially in Crohn’s disease). Iron deficiency anemia (IDA) occurs when iron stores are fully depleted and iron supply is insufficient for erythropoiesis [5,6,7].

Anemia causes multiple symptoms including fatigue, hair loss and increased susceptibility to infection, and is additionally associated with impaired quality of life and increased hospitalization rates and lengths of stay in patients with IBD. Iron deficiency (ID), even without anemia, can be debilitating, affecting overall health and daily work and social life. It is also associated with stunted growth and cognitive deficits [8,9,10,11]. Thus, it is important not only to treat patients with anemia, but also to pre-empt anemia in those with advancing ID. While IDA is well defined and generally easy to detect, ID screening is more challenging, despite a plethora of iron status parameters. Serum ferritin (SF) provides an indirect estimate of body iron stores and is the most specific biomarker of AID. However, in patients with IBD, the acute-phase response induced by acute inflammation and/or infection can elevate ferritin levels independent of iron stores, thus obscuring the presence of AID. In addition, patients with active IBD may have inflammation-induced anemia, known as anemia of chronic disease (ACD) [12,13], characterized by apparently normal or increased iron stores but limited iron availability for erythropoiesis (iron-restricted erythropoiesis; IRE). Furthermore, the fact that these two types of anemia, IDA and ACD, often occur in combination, complicates not only the determination of clinical status, but also decision-making in anemia management in these patients [14,15].

The search for laboratory tests that—unlike conventional approaches based on serum ferritin (SF) and transferrin saturation (TSAT)—provide a reliable diagnosis of iron deficiency independent of inflammation is ongoing [16]. In their “European consensus on the diagnosis and management of ID and anemia in IBD”, the European Crohn’s and Colitis Organization (ECCO), suggested using additional biomarkers (e.g., CHr, %Hypo) to distinguish functional iron deficiency from absolute iron deficiency in ACD [15].

Zinc protoporphyrin (ZnPP), reported by Dagg et al. as early as 1966 as a potential indicator of ID [17], is a highly sensitive biomarker of iron availability during erythro-poiesis [18]. When iron supply for erythropoiesis falls to a suboptimal level, zinc, instead of iron, is incorporated into protoporphyrin IX, and thus ZnPP is produced instead of heme. If the supply of iron for erythropoiesis is insufficient or iron utilization is impaired (e.g., in anemia of chronic disease), zinc is used in the biosynthetic pathway of heme instead of iron, resulting in iron–zinc substrate competition for ferrochelatase and the formation of ZnPP. Thus, ZnPP levels are a direct marker of iron status in the bone marrow during erythropoiesis [18,19,20]. ZnPP production is entirely unaffected by ACD or chronic inflammation and is therefore an effective indicator of ID even in the presence of inflammation [20,21]. The onset of iron-deficient erythropoiesis triggers continuously increasing ZnPP concentrations. Measurement of ZnPP concentration provides a reliable index of FID and may be used as an alternative to indices of red cell hypochromia or reticulocyte hemoglobin content. ZnPP has been suggested to detect inadequate iron supply in various different adult populations [22,23,24,25] and in children [26,27].

Despite its potential clinical utility for the assessment of iron status in the context of inflammation [22,24,28], only one study has evaluated ZnPP in patients with IBD, in a pediatric population [26]. However, the study included a relatively small number of patients, the majority of whom had a diagnosis of Crohn’s disease (CD). We therefore aimed to investigate the diagnostic performance of ZnPP as a marker of iron-restricted erythropoiesis in adult patients with IBD, including individuals with both CD and ulcerative colitis (UC).

## 2. Materials and Methods

We conducted a cross-sectional study using routine blood samples from patients with IBD consecutively attending regular follow-up consultations as outpatients at the Interdisciplinary Crohn Colitis Center (ICCC) Rhein-Main, Frankfurt am Main, Germany. All patients identified as suitable for study participation were comprehensively informed about all aspects of the study and required to give written informed consent prior to data extraction and analysis. The study was approved by the local ethics committee of the Ärztekammer Hessen (2019-1317-evBO; 13 February 2020). Blood sample analysis included markers of inflammatory activity, anemia and iron status. In addition, data describing the underlying diagnosis, clinical characteristics, comorbidities, treatments and drug doses were retrieved from the patients’ medical records.

The inclusion criterion was a verified diagnosis of IBD according to ECCO criteria [15]. Exclusion criteria were incomplete blood samples, history of any surgery or admission to hospital within one month prior to study entry, inherited blood disorder (thalassemia trait, hemoglobinopathies), history of zinc deficiency (serum zinc < 0.66 µg/mL) or supplementation within the previous three months, history of transfusion or iron supplementation within the previous three months and current pregnancy.

Blood samples were collected at 8 a.m. after 12 h/overnight fasting and analyzed for standard blood count, iron status (SF, TSAT), inflammation parameters (high-sensitivity C-reactive protein; hsCRP) and ZnPP. Serum and EDTA monovettes were centrifuged and then stored for further processing. Blood count was measured using the Sysmex^@^ XS-100i hematology analyzer (Sysmex Deutschland GmbH, Norderstedt, Germany), while SF, iron and hsCRP were determined using the COBAS Integra^®^ 400 plus analyzer (Roche Diagnostics Deutschland GmbH, Mannheim, Germany). ZnPP was measured fluorometrically (Fluometer 206 M; Avivi Biomedical Inc, Lakewood, NJ, USA), and TSAT was calculated from iron and transferrin values.

Evaluation of the analytical performance of iron status biomarkers to detect IRE requires a priori identification of the affected patients. While analysis of iron supply in the bone marrow with Perls’ Prussian blue staining is the “gold standard” for the diagnosis of ID [29], this technique is costly, highly invasive and nonautomated, and is therefore not practicable in patients with IBD in routine practice. Since the use of a single iron status parameter could result in over- or underdiagnosis of IRE, a multiparameter index test was performed [30,31]. Patients were assigned to the “IRE group” if at least two of three parameters (TSAT, SF, ZnPP) indicated IRE, or the “no IRE group” if a maximum of one parameter was positive [22]. Cut-off values for TSAT (<20%) and SF (<30/µL) were defined according to a consensus statement [6] and the ECCO guideline [15]. The cut-off value for ZnPP (40 µmol/mol hemoglobin (Hb)) was defined according to the literature [19,20,24,32].

Criteria taken to indicate AID were hsCRP <5 mg/L, SF 30 µg/L and TSAT < 20%, while FID was denoted by hsCRP ≥ 5 mg/L, SF < 100 µg/L and TSAT < 20% [6,15]. Anemia was defined according to World Health Organization (WHO) criteria [33] as Hb below 13 g/dL for males and 12 g/dL for females, and subclassified as IDA when AID and anemia were present, as ACD in patients with evidence of chronic inflammation (hsCRP ≥ 5 mg/L), ferritin > 100 mg/L and TSAT < 20% and as ACD/IDA where there was evidence of inflammation (hsCRP ≥ 5 mg/L) together with ferritin 30–100 mg/L and TSAT < 20% [34].

Statistical analyses were performed for patients with Crohn’s disease and ulcerative colitis, respectively, using IBM SPSS Statistics 24 (SPSS Inc. an IBM, Armonk, NY, USA) and Microsoft Excel (Microsoft Office 365, Microsoft Corporation, Redmond, WA, USA). The Kolmogorov–Smirnov test was used to assess the datasets for normal distribution.

Quantitative parameters with normal distribution were described by mean and standard deviation (SD), while abnormally distributed parameters were described by minimum and maximum, median values and quartiles. Absolute and percentage frequencies were used to describe nominal values. Parametric (Student’s *t*-test, ANOVA test) and nonparametric tests (Mann–Whitney U test, Kruskal–Wallis test) were used according to the different hypotheses to compare the groups. In addition, receiver operating characteristic (ROC) analyses were used to define the diagnostic performance of different parameters to detect IRE. The significance level was predefined at 5% (*p* < 0.05) in all tests performed.

## 3. Results

### 3.1. Baseline Patient Characteristics

Data from 130 patients with IBD (51 female, 79 male; 82 CD, 48 UC) aged 18–65 years, with a mean age (±SD) of 37.3 ± 11.8 years, were included in the study. The sample characteristics and laboratory baseline values of all patients are described in Table 1.

### 3.2. IRE Status of Patients

**Figure 1:**
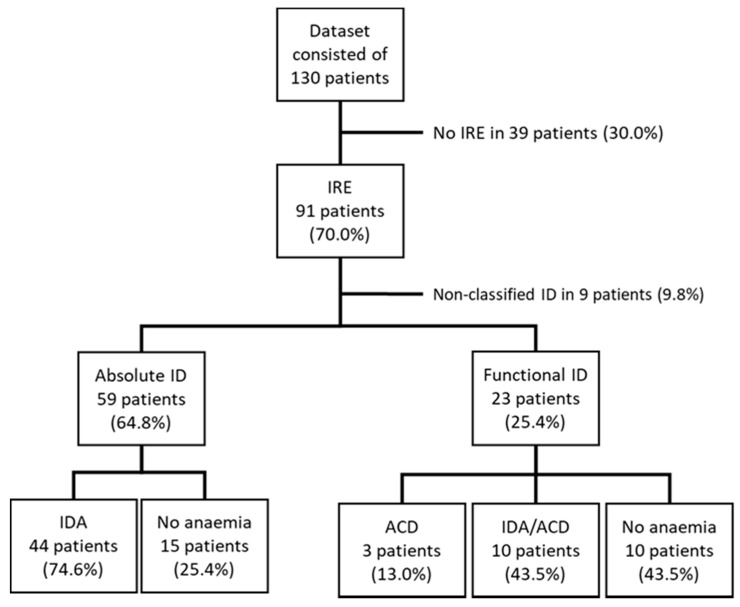
Prevalence of iron deficiency and anemia in patients with inflammatory bowel disease (IBD). Data are expressed as numbers (with percentages). ACD, anemia of chronic disease; ID, iron deficiency; IDA, iron deficiency anemia; IRE, iron-restricted erythropoiesis.

Overall, 91/130 (70.0%) of the patients had IRE. Of these, according to the laboratory markers, 59 (64.8%) had AID, while 23 (25.4%) had FID. Nine patients (9.8%) did not meet any of the pre-defined criteria and were thus categorized as having non-classified ID. Of the patients with AID, 44/59 (74.6%) met the criteria for IDA, while 3/23 (13.0%) of the patients with FID met the criteria for ACD, and 10/23 (43.5%) for mixed IDA/ACD. Thus, 15/59 (15.4%) in the AID group and 10/23 (43.5%) in the FID group had ID without anemia (Figure 1). Laboratory markers of the patients according to their iron status are summarized in Table 2 (see also Supplementary Table S1, study data sheet).

### 3.3. Analytical Performance of ZnPP as a Marker of IRE

ROC analysis was performed to compare different parameters as detectors of IRE, and to summarize the performance of each parameter as a sole marker (Table 3). As sole biomarkers, compared with SF and mean corpuscular volume (MCV), both TSAT and ZnPP, respectively, demonstrated a superior diagnostic performance in the detection of IRE (area under curve (AUC) values; 77.2%, 76.3%, 83.4% and 85.5%, respectively). However, in comparison to TSAT, the specificity of ZnPP as a marker of IRE was higher (51% vs. 70%, respectively), while both showed a similar sensitivity (97% vs. 98%, respectively).

In patients without inflammation, the findings were similar overall, with one difference; in this instance, the specificity of TSAT was slightly higher than that of ZnPP (73% vs. 67%, respectively), while both TSAT and ZnPP again showed a better diagnostic performance than MCV and ferritin (AUC values; 88.8%, 84.4%, 78.1% and 71.2%, respectively).

In the presence of inflammation, compared with MCV, TSAT and SF, ZnPP was found to be the most reliable sole biomarker for the detection of IRE (AUC values; 86.2%, 76.2%, 81.8% and 76.9%, respectively). Although the AUC of TSAT was high, its specificity was significantly lower than that of ZnPP in the presence of inflammation (38% vs. 71%, respectively).

## 4. Discussion

Iron deficiency anemia is one of the most common complications and extraintestinal manifestations of IBD and can substantially impact patients’ quality of life [35]. In IBD, IDA is the most common type of anemia, followed by ACD. Many patients with IBD have mixed anemia (IDA/ACD), in which these two types overlap [36]. Since different types of anemia require different treatment approaches, it is critical to identify which type of anemia is present in each individual case. Especially in patients with coexisting inflammation, the classification of anemia remains problematic. Moreover, there is growing evidence that an adequate iron supply is essential regardless of whether anemia occurs; iron deficiency merits treatment in nonanemic patients to maintain quality of life, and could also substantially reduce the cost of patient care. Clinically, therefore, it would be helpful to identify treatment response through early changes in erythropoiesis, even before an increase in hemoglobin is evident [8,15,37], thus avoiding delays in assessing the effects of the selected therapy. This necessitates a sensitive estimation of bone marrow response to iron replacement. However, the differential diagnosis between IDA, ACD and ACD with associated IRE is challenging [14,38]. Since a large proportion of patients diagnosed with ACD evidently respond to parenteral iron, it seems possible that iron deficiency has causal involvement in ACD. Therefore, by differentiating the causal diagnosis in ACD, a treatable subgroup of patients with ACD can be identified. This may be particularly advantageous in patients with IBD scheduled for surgery, allowing for preoperative optimization over and above the immediate treatment of the chronic illness [15].

Of all the investigated parameters, ferritin was the poorest marker of IRE, for which there are likely several reasons: First, ferritin is an acute phase protein and therefore affected by inflammation, which complicates the interpretation of concentration fluctuations [4,36]. In inflammation, serum ferritin may be “erroneously normal”, i.e., its value may be consistent with those in the general population, in spite of inadequate total iron supply. Second, ferritin is not sensitive to IRE, being a marker of body iron stores. Hence, values below the cut-off do not necessarily indicate IRE, as iron supply may still be adequate (e.g., through the phagocytosis of old erythrocytes) [16].

Although TSAT significantly outperformed ferritin in detecting IRE, it still misclassified many patients. TSAT shows substantial biological instability, not least because serum iron levels—used to calculate TSAT—show diurnal fluctuation and are influenced by oral iron supplements and the amount of iron in the diet. To minimize variation, serum iron should generally be measured in the morning after overnight fasting. Serum iron measurements are, however, not only subject to physiological variability, but also to a degree of interassay variability, casting further doubt on the accuracy of TSAT levels [39,40].

In recent years, a variety of parameters have been studied for their utility as indicators of true iron deficiency in association with inflammation. One of these laboratory parameters is the serum concentration of soluble transferrin receptor (sTfR), which is an indicator of iron requirements for iron erythropoiesis. However, cellular transferrin receptor expression is also influenced by inflammation, which may negatively affect the sensitivity of sTfR levels to indicate true iron deficiency in the presence of inflammation [41]. The calculated ratio, transferrin receptor:log10ferritin (TfR-F), has been suggested to be a more reliable marker, and has been shown to offer superior discrimination compared with either sTfR or ferritin alone, particularly in patients with chronic inflammatory disease [42,43,44]. However, TfR-F has limited availability and cost constraints [45].

The measurement of hepcidin has also been proposed as a possible tool in this context. Hepcidin directly reflects the mechanism controlling iron homeostasis. It can therefore be used as a predictor of favorable response to oral iron treatment in patients with IBD [46] and enables clinicians to design optimal oral iron dosing and timing schedules that exploit conditions minimizing the iron-triggered induction of hepcidin [47]. Hepcidin is also useful in the diagnosis of concomitant iron deficiency in patients with ACD in rheumatoid arthritis and IBD [48,49]. However, antibodies used in the immunoassays can cross-react with biologically inactive hepcidin, as a result of which true bioactive hepcidin is overestimated [50]. Although several assays have been developed, a gold standard is still lacking. Efforts towards harmonization are ongoing.

Another diagnostic marker with the potential to determine iron availability during erythropoiesis is ZnPP, which primarily forms when iron supply to the erythron is insufficient or functionally impaired. In this situation, instead of ferrous iron, divalent zinc is incorporated into the heme scaffolding [19,20,22,26]. It has been questioned whether zinc deficiency, which is not uncommon in patients suffering from IBD-related diarrhea, may have some influence on ZnPP levels. However, this possibility has not been investigated to date. In our study, all patients were screened for zinc deficiency (defined as a serum zinc measurement < 0.66 µg/mL) prior to study inclusion.

The data presented in the current analysis support the reliability of ZnPP as a detector of IRE. ZnPP values were found to be higher in AID than FID, in line with a previous publication [19]. Although this difference was not statistically significant, it seems clinically relevant and would justify further research in an even larger patient population. Our results suggest that ZnPP may be unaffected by inflammation, unlike other iron markers such as SF and TSAT, which are increased in the presence of inflammation (FID, ACD and ACD/IDA) but sensitive enough to address AID. Thus, ZnPP was successful in specifically detecting IRE, regardless of the presence of inflammation.

Our study has a few limitations: First, we had no data on soluble transferrin receptor (sTfR), since this measure is not included in our routine tests for iron status assessment. Second, due to the retrospective character of the study, no information was available on symptoms and signs of clinical activity or disease duration at the time of blood sampling.

The main strength of our study was the inclusion of a large population of patients with IBD, allowing us to further classify the patients into subgroups using a multicriterion model. As a further highlight, we were able to include patients with active inflammation—in contrast to previous studies which excluded patients with elevated inflammation parameters, thus neglecting possible effects in relation to FID, ACD and IDA/ACD. To the best of our knowledge, therefore, ours was the first study in adults with IBD and the first to focus on the effects of inflammation on ZnPP levels in this patient population. Based on our data, further studies should be performed to identify the most accurate method of detecting inadequate erythropoiesis.

## 5. Conclusions

Our data indicate that ZnPP could be a reliable marker of iron status in patients with IBD, especially when combined with other iron status parameters. Furthermore, we suggest the use of ZnPP not only in IBD but also in patients with other chronic inflammatory diseases. However, the exact determination of iron status in patients with IBD continues to be challenging. Further research is therefore needed to confirm our results and improve the understanding of iron status, iron biomarkers and iron replacement therapy in patients with chronic inflammatory disease.

## Figures and Tables

**Table 1:** Baseline patient characteristics.

Characteristic	All Patients	Crohn’s Disease	Ulcerative Colitis	*p*_1_	*p*_2_
*n* (♀)	130 (51)	82 (31)	48 (20)	-	-
Age (years), mean ± SD	37.3 ± 11.8	37.5 ± 13.0	36.8 ± 9.3	-	0.992
Disease localization, *n* (%)					
E1 proctitis	5 (3.8)	-	5 (10.4)	-	-
E2 left sided colitis	31 (23.8)		31 (64.6)		
E3 pancolitis	12 (9.2)		12 (25.0)		
L1 ileum	44 (33.8)	44 (53.7)	-	-	-
L2 colon	29 (22.3)	29 (35.4)			
L3 ileocolic	9 (6.9)	9 (11.0)			
Laboratory markers, median (min–max)					
Hb (mg/dL)	12.0 (5.7–16.2)	12.1 (5.7–15.6)	11.6 (8.0–16.2)	-	0.184
MCV (fl)	86.0 (28.5–226.0)	85.8 (55.1–118.8)	86.8 (28.5–226.0)	-	0.595
SF (mg/L)	25.0 (2.0–709.0)	33.4 (2.0–709.0)	15.8 (6.0–525.0)	-	0.027 *
TSAT (%)	11.6 (2.0–88.6)	11.0 (2.0–88.6)	12.2 (2.8–47.5)	-	0.390
ZnPP (µmol/mol Hb)	56.8 (24.5–169.1)	58.7 (24.5–169.1)	55.7 (28.8–145.1)	-	0.993
hsCRP (mg/L)	5.10 (0.3–122.9)	7.0 (0.4–108.0)	2.7 (0.3–122.9)	-	0.011 *

* *p* < 0.05. *p*_1:_ chi-square test, *p*_2:_ Mann–Whitney U test; Hb, hemoglobin; MCV, mean corpuscular volume; SF, serum ferritin; TSAT, transferrin saturation; ZnPP, zinc protoporphyrin; hsCRP, high-sensitivity C-reactive protein.

**Table 2:** Laboratory markers according to iron status.

Parameter	Absolute ID (*n* = 59)	Functional ID (*n* = 23)	*p*	IDA (*n* = 44)	ACD or IDA/ACD (*n* = 13)	*p*
Hb (g/dL)	11.5 (5.7–11.6)	12.3 (9.2–15.2)	0.005 *	10.8 (5.7–12.9)	11.8 (9.2–12.9)	0.004 *
MCV (fl)	82.8 (28.5–226.0)	85.5 (76.2–98.2)	0.071	82.2 (55.1–226.0)	85.0 (76.2–98.2)	0.106
SF (µg/L)	11.3 (2.0–29.6)	53.7 (30.8–328.0)	0.000 *	9.8 (2.0–27.3)	47.3 (30.8–275.0)	0.000 *
TSAT (%)	8.9 (2.0–32.6)	10.5 (2.2–16.9)	0.045 *	6.4 (2.0–32.6)	10.0 (6.6–16.9)	0.020 *
ZnPP (µmol/mol Hb)	66.5 (39.9–169.1)	58.7 (35.2–119.5)	0.322	66.7 (40.1–169.1)	66.7 (41.6–119.5)	0.413
hsCRP (mg/L)	2.8 (0.3–44.0)	13.3 (5.1–108.0)	0.000 *	3.3 (0.3–44.0)	15.7 (6.9–77.3)	0.000 *

* p < 0.05, Mann–Whitney U test; Hb, hemoglobin; MCV, mean corpuscular volume; SF, serum ferritin; TSAT, transferrin saturation; ZnPP. zinc protoporphyrin; hsCRP, high-sensitivity C-reactive protein. Table includes only classified anemia, patients with unclassified ID (*n* = 1) and unclassified IDA (*n* = 8) are therefore not shown.

**Table 3:** Analytical performance of iron status parameters to detect patients with IRE depending on the probability of co-presence of inflammation.

Analysis	MCV	TSAT	SF	ZnPP
All patients				
AUC^ROC^	0.763 (0.683–0.843)	0.834 (0.757–0.911)	0.772 (0.686–0.858)	0.855 (0.773–0.998)
Sensitivity	27%	97%	65%	98%
Specificity	100%	51%	72%	70%
Cut-off	80 fl	20%	30 µg/L	40 µmol/mol Hb
*p*	<0.001	<0.001	<0.001	<0.001
Patients with inflammation				
AUC^ROC^	0.762 (0.651–0.873)	0.818 (0.715–0.921)	0.769 (0.658–0.879)	0.862 (0.752–0.971)
Sensitivity	27%	100%	82%	98%
Specificity	99%	38%	42%	71%
Cut-off	80 fl	20%	100 µg/L	40 µmol/mol Hb
*p*	<0.001	<0.001	<0.001	<0.001
Patients without inflammation				
AUC^ROC^	0.781 (0.666–0.896)	0.888 (0.785–0.992)	0.712 (0.550–0.874)	0.844 (0.718–0.971)
Sensitivity	30%	93%	80%	98%
Specificity	100%	73%	46%	67%
Cut-off	80 fl	20%	30 µg/L	40 µmol/mol Hb
*p*	<0.001	<0.001	0.014	<0.001

AUC^ROC^, area under curve receiver operating characteristic; MCV, mean corpuscular volume; TSAT, transferrin saturation; SF, serum ferritin; ZnPP, zinc protoporphyrin.

## Data Availability

The data underlying this article are available in the article and in its online supplementary material.

## Supplementary Materials

The following are available online at https://www.mdpi.com/2075-4418/11/2/366/s1, Table S1: Study data sheet.

## Abbreviations

ACD	anemia of chronic inflammation
AID	absolute iron deficiency
AUC	area under curve
CD	Crohn’s disease
CRP	C-reactive protein
ESR	Erythrocyte sedimentation rate
FID	functional iron deficiency
Hb	hemoglobin
hsCRP	high-sensitivity C-reactive protein
%HYPO	hypochromic red blood cells
IBD	inflammatory bowel disease
ID	iron deficiency
IDA	iron deficiency anemia
IRE	iron-restricted erythropoiesis
IV	intravenous
MCHC	mean corpuscular hemoglobin concentration
MCV	mean corpuscular volume
ROC	receiver operator characteristic
SD	standard deviation
SF	serum ferritin
sTfR	soluble transferrin receptor
TSAT	transferrin saturation
UC	ulcerative colitis
ZnPP	zinc protoporphyrin
